# No evidence for deleterious effects of psilocybin and MDMA on working memory or related neurophysiological activity in a non-clinical population: An uncontrolled exploratory study

**DOI:** 10.1177/02698811261464991

**Published:** 2026-07-23

**Authors:** Neil Wayne Bailey, Samantha Lee Webb, Bernadette Mary Fitzgibbon, Nigel Christopher Denning, Traill Dowie, Monica Margaret Schweickle, Anish Modak, Grace Chan, Jackie Knight, Molly Waldron, Kirsten Gainsford, Hannah Hawkes, Sonia Zammit, Novi Christina Sutanto, Kate Godfrey, Paul Bernard Fitzgerald

**Affiliations:** 1 Cognitive Neuroscience Unit, School of Psychology, Deakin University, Burwood, VIC, Australia; 2 School of Medicine and Psychology, The Australian National University, Canberra, ACT, Australia; 3 Monarch Mental Health Group, Sydney, NSW, Australia; 4 Integrative Psychology and Medicine, Melbourne, VIC, Australia; 5 La Trobe University, Melbourne, VIC, Australia; 6 Ikon Institute of Australia, Melbourne, VIC, Australia; 7 Peridot Clinic, Newcastle, NSW, Australia; 8 Department of Brain Sciences, Imperial College London, UK

**Keywords:** psilocybin, MDMA, psychedelics, working memory, cognition, neural activity, electroencephalography

## Abstract

**Background:**

Research indicates psychedelics hold therapeutic potential, but possible deleterious effects have been insufficiently examined. We explored working memory (WM) performance and WM-related neurophysiological activity before and after exposure to psilocybin or 3,4-methylenedioxymethamphetamine (MDMA) in an uncontrolled study.

**Methods:**

Healthy participants were exposed to a single dose of psilocybin or MDMA (data analysed from 29 participants for each drug, with 16 receiving both drugs after >3-month washout). One to 16 days before and 5–16 days after dosing, participants completed a 3back WM task and eyes-closed resting with electroencephalography (EEG), plus a 3back during a 3-month remote follow-up.

**Results:**

After dosing, both groups showed increased alpha to gamma WM-related oscillatory power (*p* < 0.001). After psilocybin, participants showed steeper WM-related aperiodic slopes (*p* = 0.018), an effect maximal in occipital electrodes (*p* < 0.001, Cohen’s *d* = 0.802, BF_10_ = 155.138), and significant in occipital electrodes during resting (*p* = 0.008). The MDMA group showed improved task accuracy in the post-dosing EEG session (*p* = 0.005, Cohen’s *d* = 0.561, BF_10_ = 7.809). Both groups showed improved accuracy at 3-months (*p*-holm < 0.001, Cohen’s *d* = 0.678, BF_10_ = 272.074).

**Conclusions:**

Our results are suggestive of enduring neurophysiological effects following a single dose of MDMA or psilocybin. Our results do not support concerns about cognitive impairments from single exposures to MDMA or psilocybin in controlled settings, suggesting clinical utility does not risk impairing WM. However, given the uncontrolled study design, future research is required to confirm our observations reflect drug-induced neurophysiological changes.

## Introduction

After decades languishing under prohibitionary regulations, psychedelic substances have re-emerged as potential therapies for selected psychiatric conditions. In particular, psilocybin (a serotonergic tryptamine with a primary mechanism of action as a 5-HT2A receptor agonist) has shown promise as a treatment for depression (Gukasyan et al., 2022; Metaxa and Clarke, 2024), while 3,4-methylenedioxymethamphetamine (MDMA—a serotonin–norepinephrine–dopamine releasing agent and serotonin–norepinephrine–dopamine reuptake inhibitor) has shown promise for treating post-traumatic stress disorder (Illingworth et al., 2021; Shahrour et al., 2024). The therapeutic effects of psilocybin have been suggested to be related to the ability of the substance to disrupt rigid cortical network activity and increase neuroplasticity, which may enable new neural activity pathways and related cognitions to develop in patients (Prouzeau et al., 2022). Similarly, the therapeutic effects of MDMA are theorised to relate to an ability to re-perceive previous traumatic experiences with a reduced fear response, which may be driven by MDMA’s ability to increase brain-derived neurotrophic factor in fear memory learning pathways, as well enhancement of therapeutic engagement and rapport due to the drug’s pro-social effects (Sottile and Vida, 2022). However, more research is required to properly understand the mechanisms and effects of these drugs.

Acute administration of psilocybin has been associated with a marked decrease in alpha oscillatory activity, as well as oscillatory activity in other bands (an effect that is relatively consistent across serotonergic psychedelics; Godfrey et al., 2025). These characteristic changes in neural activity may relate to the subjective effects of the drug, with the disrupted alpha activity related to altered default mode activity, proposed to reflect a reduction in the engagement of fixed cognitions that may underlie typical ruminative thought patterns in depression (Nikolic et al., 2024). Similarly, acute MDMA administration is associated with reduced alpha and theta activity, and with an increase in high frequency (gamma) power (Frei et al., 2001; Lansbergen et al., 2011). In addition to likely effects on neural oscillations, reductions in low frequency power and increases in high frequency power are suggestive of a flattening of the aperiodic activity slope during acute administration, a finding that has been reported for several other psychedelics (although not MDMA or psilocybin specifically as far as we are aware; Godfrey et al., 2025). This effect during acute exposure may reflect a shift towards increased excitatory tone from excitatory effects related to the increases in serotonin, norepinephrine, and dopamine signalling.

However, although these acute mechanisms are becoming more extensively studied and understood, the non-acute effects of these drugs are not well characterised, with only one study that we are aware of currently having compared electroencephalography (EEG) measures between pre- and post-exposure beyond the 24-hour mark for psilocybin (Skosnik et al., 2023), and no research having done so for MDMA. Improving our understanding of the non-acute effects is critical for determining their safe use in clinical applications, and for understanding the mechanisms of action by which ongoing clinical effects persist. These points are particularly important for MDMA, with previous research into recreational use of the drug having suggested it is associated with executive functioning and working memory (WM) updating impairments (Montgomery and Roberts, 2022; Roberts et al., 2016). Thus, confidence that clinical applications of MDMA are likely to provide a net benefit requires examination of not only the potential benefits of the drug when combined with therapy but also an assessment of any potential harms. In contrast, psilocybin has been suggested to improve a range of cognitive functions, a prospect that, if robust, may hold promise to address cognitive impairments in depression, as well as mood symptoms (Meshkat et al., 2024).

To help provide a better understanding of these points, the current study aimed to explore potential changes in WM and executive function performance, as well as related neural activity from before to after a single exposure of either psilocybin or MDMA in a non-clinical sample. In this study, we used the 3back WM task, which presents sequences of stimuli (often letters) and requires participants to press a button if the currently presented stimulus is the same as the stimulus presented three presentations earlier. Thus, the 3back task requires WM and executive function to remember each stimulus and constantly update the relevant letter sequence in memory. The 3back task is subjectively difficult for most participants, with a typical mean percentage of correct responses of 80–90% (Bopp and Verhaeghen, 2020), providing good sensitivity to detect changes in WM or executive function performance.

A wide range of oscillatory frequencies have been suggested to be relevant for WM functions. Theta oscillatory power (4–8 Hz) has been suggested to relate to cognitive control functions, the allocation of cognitive resources to completing task demand-related processes (Sauseng et al., 2010), and/or the neural activities that enable temporal organisation of WM stimuli (Roux and Uhlhaas, 2014). Participants with better WM performance have been reported to show larger theta increases for higher WM loads (Dong et al., 2015; Gevins and Smith, 2000). Gamma oscillatory power (30–80 Hz) increases have been suggested to underpin the intensive cognitive processing performed within specific brain regions to encode and rehearse WM stimuli (Roux and Uhlhaas, 2014; Tallon-Baudry et al., 1998). Increases in beta (14–30 Hz) and decreases in posterior alpha (8–13 Hz) oscillatory activity have also been reported for higher WM loads (Chen and Huang, 2016). The decreases in alpha power may be reflective of alpha’s putative function as a top-down inhibition signal that enables suppression of processing in non-task-relevant brain regions to improve the signal to noise ratio of relevant processes (Klimesch, 2012). However, in the 3back task, higher alpha power has also been reported to be associated with faster correct response times (Chung et al., 2018). In contrast, beta has been suggested to underpin network connectivity and the re-activation of internal representations of information from memory (Spitzer and Haegens, 2017).

In addition to the oscillatory activity associated with the 3back task, recent research has suggested that the steepness of the slope of the overall frequency power spectrum (after excluding the influence of oscillatory activity) is important in WM (Virtue-Griffiths et al., 2025; Voytek et al., 2015). This non-oscillatory aperiodic activity typically shows a log-frequency log-power slope of approximately 1/*f*, where power decreases as frequencies increase (Voytek et al., 2015). Modelling work shows that the overall shape of this 1/*f* activity is driven by a fundamental limitation of the recording of EEG signals. Computational models indicate that for neurophysiological activity generated by propagating cortical spikes to be of a sufficient amplitude to be detectable in scalp EEG recordings, their signals must show higher power at low frequencies compared to higher frequencies (Brake et al., 2024). The exact slope of this aperiodic spectrum activity has been suggested to be reflective of variation in excitation/inhibition balances (Gao et al., 2017). However, we note that recent research suggests the aperiodic slope provides only an inconsistent measure of the excitation/inhibition balance that is dependent on neuronal conductance leakage (Brake et al., 2024). The slope has also been suggested to be modified by variations in synaptic kinetics and aperiodic network dynamics, including long-term autocorrelations (Brake et al., 2024). Irrespective of the exact neurophysiological drivers of the aperiodic activity, steeper 1/*f* slopes have been shown to be related to higher WM capacity (Virtue-Griffiths et al., 2025).

Given this background, we hypothesised that both psilocybin and MDMA would be associated with typical practice effect-like improvements in WM performance, rather than the decreases in WM performance that might suggest impaired neural function due to the drug exposure. We also hypothesised that we would observe enhanced WM-related oscillatory activity after exposure to both drugs, in particular, enhanced theta and gamma activity, given their functional roles in the 3back task. However, given the novelty of the current data, we also explored effects in other frequencies (3–45 Hz). Finally, we had an exploratory hypothesis that we would observe changes to the slope of participants’ aperiodic activity (with a non-directional hypothesis). Unfortunately, we were unable to include a control condition within our study. Thus, it is worth the reader keeping in mind while reading this article we cannot rule out the possibility our results may be explainable by factors other than exposure to MDMA and psilocybin. Nevertheless, with the exception of this limitation, our study provides an excellent example of the methodological rigour required to study the effects of psychedelic substances on cognition and neural activity, featuring dosing sessions conducted by experienced practitioners, controlled substance quality, single-drug exposure, and elimination of polydrug confounds.

## Methods

### Study design

The primary study (“A study of the psychological, cognitive and physiological effects of Psychedelic Medicines” (the ASSESS trial)) examined changes associated with a single dose of psilocybin and MDMA drug exposure on psychological, cognitive and neurophysiological measures between 1 and 16 days pre-dosing, 5 and 16 days post-dosing, and 3-month follow-up timepoints, using an uncontrolled study design. The study was conducted in accordance with the Declaration of Helsinki and International Conference on Harmonisation Guidelines in Good Clinical Practice and the study was approved by the Human research Ethics Committees of ACT Health (2022.ETH.00184) and the Australian National University. All participants provided informed consent, and the study was registered with Australian New Zealand Clinical Trials Registry (ACTRN12622001535763p, https://anzctr.org.au/ACTRN12622001535763.aspx, date of registration: 12 December 2022).

### Participants

A total of 50 healthy individuals consented to participate in the study. Recruitment, dosing sessions, and assessments took place from 2023 to 2024 in Melbourne, Australia. One participant withdrew prior to their dosing session, and one participant was excluded on the day they were to receive the drug due to high blood pressure, leaving a total of 48 participants who were exposed to a single dose of either MDMA or psilocybin in a group setting (with between 2 and 4 participants receiving either MDMA or psilocybin during the same session—the full details of these sessions has been reported previously; Fitzgerald et al., 2025). We note that this is below our initial recruitment goal of 100 participants, but recruitment was abbreviated due to resource limitations. Due to the problematic nature of post hoc power analyses (Hoenig and Heisey, 2001), instead of power analyses, we used Bayesian statistics to provide an indication of the strength of the evidence for the alternative or null hypotheses, given the observed effect size and our sample size. Participants were permitted to select the drug they would receive in the dosing session (but all participants within a dosing session received the same drug). Participants were provided with the option to participate twice, after at least 3 months between sessions, with their second session involving the drug that they did not receive in their first dosing session.

Of the 48 participants who were exposed to 1 of the drugs, 25 initially received MDMA and 23 psilocybin. With regards to participants who participated twice, 10 participants received MDMA and then psilocybin, while 6 participants received psilocybin and then MDMA. Thus, a total of 31 MDMA and 33 psilocybin doses were administered. From these dosing sessions, a total of 29 pre and in the post-dosing EEG recordings were available for each drug. Data from two participants in the MDMA condition and four participants in the psilocybin condition were not included in the current study due to travel limitations (*n* = 3) or unexpected changes in availability (*n* = 2) that meant they could not attend both EEG sessions, or due to measurements in a final screening ruling them ineligible on the day of dosing (*n* = 1).

All participants had received training in psychedelic assisted psychotherapy. Due to this training, participants were aware of the expected effects and side effects of each drug. All participants included in this study of the 3back task were between 31 and 68 years of age (psilocybin mean = 46.79, SD = 9.32, MDMA mean = 49.59, SD = 12.00), able to provide informed consent, and were proficient in written and spoken English. All participants were required to nominate a support person who would be in the same house as the participant until the day following the dosing session. Exclusion criteria included pregnancy, unstable medical illness, a form of cardiovascular disease that could pose a risk to the participant during the dosing session, current psychoactive medication, a history of seizures or moderate to severe traumatic brain injury, hepatic impairment, and insulin-dependent diabetes. Exclusion criteria also included a current or previous diagnosis of schizophrenia spectrum or other psychotic disorder, bipolar disorder, or alcohol or other substance dependence. Participants were also excluded if they had used either MDMA or psilocybin in the 3 months prior to the study, or if they self-reported a lifetime history of more than 100 uses of either drug. A total of 19 females and 10 males participated in the psilocybin condition, and 20 females and 9 males participated in the MDMA condition. Participants had on average 21 years of education (psilocybin mean = 20.93, SD = 4.06, MDMA mean = 20.59, SD = 3.58).

Unfortunately, due to the extensive range of screening, dosing and testing sessions required by the study, we were unable to include a control arm due to resource limitations and the undue burden a control condition would place on participants, who were in general professionals with limited time available to participate in research that required extensive on site commitment.

### Study procedures

After participants provided informed consent and passed assessment for study suitability, EEG sessions were conducted at a pre-dosing baseline session (between 1 and 16 days before the dosing session) and 5–16 days after the dosing session. Online cognitive assessments (that did not include an EEG) were also undertaken at a 3-month follow-up timepoint. In addition to these measurement sessions, each participant undertook a single preparation session prior to the dosing session, where a study therapist briefed participants on what to expect in the dosing session. Participants also undertook an integration session with the same study therapist within 72 hours after the dosing session. Preparation and integration sessions were conducted by an experienced therapist either online or in person, and both sessions included all members of the same dosing session group. Participants were provided with written documents outlining the process of the dosing sessions and their expectations before and during the session. Dosing sessions were conducted in a large room designed to provide a low stimulus and calm environment. Calm and curated music was played by the therapist during these sessions. The dosing session room was set in a mental health clinic in Melbourne, Australia. Dosing sessions were conducted in groups of two to four individuals, monitored by two experienced therapists, with medical support available. Participants were permitted to sit and engage in conversation or lie quietly using eye shades. A second room was available so that participants who were undergoing distress of any form could be moved to that room for increased attention from one of the two therapists (without disrupting other participants). Blood pressure, heart rate and body temperature were measured prior to dosing and once per hour up to the first 3 hours after dosing by a trained research team member, as well as prior to discharge. Participants completed a side effects questionnaire before leaving the dosing session. All participants left the dosing session in the company of their nominated support person.

### Psilocybin and MDMA

For the psilocybin arm of the study, a single dose in capsule form of 25 mg of psilocybin was provided for a person weighing under 90 kg, or a single dose in capsule form of 30 mg was provided for a person weighing between 90 and 115 kg (no participants weighed more than 115 kg). All participants who received MDMA received an initial 80 mg dose with a supplementary dose of 40 mg provided if there were inadequate subjective effects after 1 hour (at the discretion of the lead therapist, participant, and study doctor). The supplementary additional half dose was provided to 25 of the 29 participants who were included in this study of the 3back data. We note that given the delay between the initial and supplementary MDMA dose was only 1 hour, and the post-dosing EEG session was 5–16 days later, receipt of the supplementary dose was unlikely to impose effects related to the temporal proximity between dosing and the EEG session.

Drugs were supplied through a contractual relationship with Mind Medicine Australia who sourced a supply of psilocybin from Psygen Labs Inc. (Calgary, Canada) and MDMA from Dalton Chemical Laboratories Inc. (Toronto, Canada), as well as OPTIMI Labs Inc. (Mordialloc, Australia) for a supply of both Psilocybin and MDMA. All drugs supplied were medical grade Good Manufacturing Practice (GMP) standard, with a Certificate of Analysis and GMP certification. Both drugs were imported by a licenced pharmacy and stored under the conditions determined in the appropriate government importing and storage permit regulations. Drug doses were couriered to the study site after trial medication prescription and the granting of an individual participant permit through the local health department processes.

### 3back task

A range of psychological and cognitive measures were undertaken by participants at the pre- and post-dosing EEG session, as well as a 10-minute eyes-closed resting recording. Analyses of the psychological measures has been reported separately (Fitzgerald et al., 2025), and analyses of the other cognitive measures (an emotional Stroop, Go Nogo task, and latent inhibition task) and related EEG data is in preparation to be reported separately. The current study focuses primarily on the EEG data from the 3back task, with an associated targeted analysis of the eyes-closed resting data to determine whether effects observed in the 3back task might reflect generalised neurophysiological changes following drug exposure. Our analysis also examined differences in behavioural performance between pre- and post-dosing EEG session. With regards to the task order for each recording session, the resting recording was undertaken after the emotional Stroop, Go Nogo, and latent inhibition tasks, and the 3back task was completed after the resting recording.

The 3back task included a single block, where participants were presented with a random sequence of 160 letters (A–J, with each letter presented with the same total frequency). Each letter was presented for 500 ms followed by a blank screen, with an intertrial interval of 2000 ms. The task required participants to press a button with the index finger of their dominant hand if the current letter was the same as the letter presented three letters earlier. A total of 25% of the letters were targets (repeats of the letter that was presented three letters prior, requiring a response), while other letters were non-targets (non-repeats of the letter that was presented three letters prior). Three versions of the task were developed, each with a different random order of letters (but the same target proportion and the same total number of presentations of each letter). Participants completed each of the three versions of the task across their pre-dosing, post-dosing EEG session and 3-month follow-up testing sessions, with the order of the tasks counterbalanced between individuals and across the two groups. Prior to each completion of each 3back task, participants completed a practice version of the task, which presented 21 trials of the 3back task with the same parameters as the real version of the task. The task was developed and presented using PsychoPy software (Peirce, 2007). During the pre- and post-dosing EEG sessions, PsychoPy was used to send separate triggers for targets and non-targets directly to the EEG amplifier for integration into the EEG recording, time-locked to stimulus presentation. Participant responses were made using a BBTKv2 BlackBox response box (The Black Box ToolKit Ltd., Sheffield, England), with response triggers ported directly to the EEG amplifier (Bridges et al., 2020). During the 3-month follow-up sessions, participants completed the 3back task via their personal computer at home, through an online hosted version of the PsychoPy 3back task hosted on the Pavlovia platform (Open Science Tools; Bridges et al., 2020).

### EEG recording and pre-processing

EEG data were recorded using an ANT Neuro eego™ amplifier using a 64-channel Ag/AgCl electrode Waveguard cap (with a 10-10 electrode layout). Data were recorded using the eego software at 1000 Hz with no online filters. The online reference was placed at CPz, and AFz was used as the ground electrode. Offline, EEG data were pre-processed using the RELAX v2.0.1 EEG pre-processing pipeline (Bailey et al., 2023b, 2023c, 2024), which is a MATLAB-based toolbox that implements EEGLAB (Delorme and Makeig, 2004) and FieldTrip functions (Oostenveld et al., 2011). RELAX uses these functions to automatically apply a selection of validated EEG pre-processing steps that reduce artefact influences while also protecting neural activity from distortion by the pre-processing (Bailey et al., 2023b, 2023c, 2024). To clean the data in this study, RELAX was used to first bandpass filter data from 0.5 to 80 Hz using a non-causal fourth order Butterworth filter and a Parks-McClellan notch filter at 50 Hz to reduce line noise. Next, electrodes that were severely affected by artefacts for prolonged periods were detected using PREP’s bad electrode detection approach, followed by the default moderate RELAX v2.0.1 bad electrode detection approach (Bailey et al., 2024), with two exceptions: (1) blink affected periods were only rejected if their maximum voltage shift within the epoch exceeded 12 median absolute deviations from the median across all blink affected periods (rather than 10 as per the default moderate settings), and (2) the absolute voltage threshold to define an extreme outlier was increased from 1000 to 1200 µV. These settings were adjusted from the default moderate settings due to a high proportion of data being rejected by the moderate settings for one participant who showed very large blink amplitudes. These bad electrodes were removed from the data. After the bad electrode removal step, segments of the data that were severely affected by artefacts were deleted, again using the thresholds explained above. Following these steps, data were re-referenced to the robust average reference (Bigdely-Shamlo et al., 2015), and independent component analysis using the PICARD algorithm was used to decompose the data (Frank et al., 2022). ICLabel and icablinkmetrics were both used to identify eye-movement artefacts (Pion-Tonachini et al., 2019; Pontifex et al., 2017), and components with log-frequency-log-power slopes that exceeded −0.59 were identified as muscle activity artefacts (Fitzgibbon et al., 2016). A wavelet transform was applied to the eye-movement artefacts to extract the dominant frequencies from these artefact components, and a threshold targeting method was used to subtract the wavelet transformed model of the artefact from only the periods of the eye-movement components that showed eye-movement artefacts above the threshold (Bailey et al., 2024). This targeted wavelet enhanced independent component analysis (ICA) cleaning approach has been shown to effectively reduce the eye-movement artefacts, while also preserving the neural activity signals that are present within the component (due to imperfect ICA decompositions; Bailey et al., 2024). Next, muscle activity components were low pass filtered at 15 Hz, which removes the high frequency activity that is typical of muscle artefacts, while also preserving the neural activity signals that are present within the component (due to imperfect ICA decompositions; Bailey et al., 2024). Other artefact components were not reduced, as averaging a sufficient number of trials effectively removes the influence of other artefacts on outcome measures, which is suggested to be preferable to the risk of removing the neural activity signals mixed into those artefacts since no effective method to target other artefacts currently exists (Bailey et al., 2024). Finally, data were reconstructed back into the scalp space, providing cleaned continuous data ready for epoching and analysis.

Data were then epoched for analyses from the −6 to 7 second period surrounding each stimulus trigger. Note that this long epoch was selected to enable valid computation of frequency power in low frequency ranges using wavelet transforms, but that our statistical comparisons focused on a 1 second period following each stimulus. Default RELAX v2.0.1 bad epoch rejection steps were applied to remove epochs that were still contaminated with artefacts after data cleaning. A regression baseline correction approach was then applied to control for variation in voltage amplitudes during the pre-stimulus period (which are often present due to voltage drift or other confounds; Bailey et al., 2023c, 2023d). Within this regression baseline correction approach, the −200 to 0 ms period was averaged from each epoch separately as the factor to correct. The task stimulus that each epoch was extracted from (target or non-target) was set as a factor of interest (so the baseline correction preserved variance associated with this factor), and a constant included in the regression model (with variance associated with the constant also preserved). This approach controls for potential voltage drift prior to each epoch, while still preserving experimental effects, and has been shown to be superior to the subtraction baseline correction approach (Alday, 2019; Bailey et al., 2023c, 2023d).

### Outcome measure computation

Following these pre-processing steps, we assessed time-resolved frequency power in epochs that were associated with correct non-target stimuli using Morlet wavelet transforms using the ft_freqanalysis Fieldtrip function. Selection of the non-target stimuli only prevented results from being confounded by behavioural responses. Frequency power was computed with a 5 ms resolution across a 3–45 Hz range (0.5 Hz steps) using five-cycle wavelets. The post-stimulus window (0–1000 ms) was baseline corrected to the −650 to −350 ms pre-stimulus period using a relative change method (active/baseline), recommended by previous research to control for the influence of ongoing frequency power (both oscillatory and non-oscillatory) that may not relate to the processing of WM stimuli, while also avoiding the smearing of post-stimulus activity into the baseline period (Chung et al., 2018). Finally, values obtained from frequency power computations were separately averaged within each participant to enable analyses.

Additional exploratory analyses were conducted to assess potential differences in the 1/*f* aperiodic slope and offset using the *fooof* application of the *ft_freqanalysis* Fieldtrip function (Donoghue et al., 2020). For this analysis, data were re-epoched to the 500 to 900 ms period following the stimuli (as our baseline corrected frequency power comparisons suggested a likely effect during this time period). The *mtmfft* setting was applied, frequencies from 1 to 45 Hz were included, padding of 4 seconds was used, and the width of frequency smoothing was set to 2 Hz. We also analysed oscillatory power within this time period, after subtracting the aperiodic power spectrum, which has been suggested to provide a measure of true oscillatory activity (which is reflected by the frequency power that sits above the aperiodic power spectrum; Donoghue et al., 2020).

To assess whether the changes in oscillatory power and aperiodic slope might reflect generalised changes or task specific changes, we also assessed oscillatory power from eyes-closed resting EEG recordings. For these analyses, we extracted 2 second epochs from the eyes-open resting data (with a 50% overlap for each epoch), then computed the oscillatory power (after controlling for the aperiodic activity) and aperiodic slope and offset for these epochs in the same way as for our 3back data.

For behavioural data, task accuracy was measured using the parsimonious accuracy measure proposed by Bustamante et al. (2006). This measure provides an unbiased combined measure of the proportion of both correct responses and correct non-responses relative to incorrect responses and non-responses. Accuracy was computed using the following method: [0.5 × the proportion of correctly responded targets relative to total target presentations] + [0.5 × the proportion of non-targets that participants correctly did not respond to, relative to total non-target presentations] (Bustamante et al., 2006).

### Statistical tests

For behavioural data, parametric frequentist and Bayesian statistical analyses were performed using JASP v0.95.1.0 (Love et al., 2019). For our primary comparisons, statistical tests of our behavioural data were performed by comparing the pre- and post-dosing EEG sessions using paired samples *t*-tests for each drug separately. Effect sizes are reported using Cohen’s *d* for paired samples *t*-tests, where effect sizes are considered to be: 0.2 = small, 0.4 = medium, and 0.6 = large. Additionally, an exploratory repeated measures ANOVA was conducted including both drugs and all sessions (with the reduced sample size due to a reduced response rate to the 3-month follow-up measurement). Partial omega squared (ω^2^_*p*_) is reported as the effect size for repeated measures ANOVAs, where effect sizes are considered to be 0.01 = small, 0.06 = medium, 0.14 = large. To provide an indication of the strength of the evidence for either the alternative or null hypotheses given the observed effect size and sample size, we also provide Bayes factor (BF) values. BF_10_ indicates support for the alternative hypothesis, with values between 1 and 3 indicating anecdotal evidence for the alternative hypothesis, values between 3 and 10 indicating moderate evidence, values between 10 and 30 indicating strong evidence, and values greater than 30 indicating very strong evidence; reciprocally, BF_10_ values below 1 indicate evidence in favour of the null hypothesis, with values below 0.33 indicating moderate evidence for the null and values below 0.10 indicating strong evidence for the null (Jeffreys, 1998; Wagenmakers et al., 2018).

For tests of neural oscillations, permutation statistics were computed in MATLAB v2025a (MathWorks, Natick, MA, USA). To assess the potential significance of power measures at each frequency and each timepoint in the epoch, we used both root mean squared (RMS), and RMS normalised topographical ANOVA (TANOVA) permutation statistical tests. These primary tests were performed separately for each frequency and timepoint, followed by cluster-based statistics to control for multiple comparisons across frequencies and timepoint. To achieve these analyses, first, we combined all frequency power timepoints and frequencies into two dimensional matrices for each participant. We then computed the RMS across electrodes, and an RMS normalised global map dissimilarity values to enable RMS and TANOVA permutation statistical tests. The RMS test involved computing the RMS of the baseline corrected frequency power across all electrodes prior to the permutation statistical testing, providing a measure of differences in global power (across all electrodes) for each frequency and timepoint (Bailey et al., 2020, 2023a). The normalised TANOVA involved first applying an RMS normalisation for global amplitude (across all electrodes) to each frequency and timepoint, which normalised all data to equal variance across all electrodes for each participant. This enabled the normalised TANOVA to test for differences in the topographical distribution of frequency power across the scalp, independently of differences in the global strength of the responses (Koenig et al., 2011). This normalised TANOVA test can indicate, for example, that although global alpha power may be the same between two conditions, one condition may show a shift towards an increased relative contribution of frontal alpha power and reduced contribution of posterior alpha power to the global alpha power (McQueen et al., 2024). After this normalisation, we computed the mean difference in frequency power between the pre- and post-dosing EEG session timepoints at each electrode, followed by computation of the RMS of this difference topography (a measure known as the global map dissimilarity). This measure indexes the strength of differences in the topographical distribution of activity between the timepoints, independent of global differences in neural response strengths (Koenig et al., 2011). Since the RMS and global map dissimilarity measures are single values, this approach eliminates the need to control for multiple comparisons across electrodes.

Following computation of these measures, we used permutation statistics to test for statistical differences in these measures across all timepoints and frequencies, while protecting against false positive inflation due to multiple comparisons. To achieve this, both the group and pre/post labels were shuffled 5000 times, and the RMS and global map dissimilarity values were computed for each of these shuffles (and the real data) at each timepoint and frequency. We then rank ordered the real effects against these permutation effects to determine the proportion of effect sizes within the real data at each timepoint and frequency that exceeded the effect sizes in the shuffled data, with *p* < 0.05 deemed significant at this primary statistical test level. We then determined the size of clusters of adjacent timepoints and frequencies that exceeded our *p* < 0.05 primary threshold, for both the real data and each of the null permutations. Then, to control for multiple comparisons across both time points within the epoch and each frequency, we determined the proportion of real cluster sizes that were larger than 95% of the clusters from the shuffled (null) data. For any significant clusters, we planned post hoc tests to explore the global neural response strength or distribution-based drivers of significant effects.

A similar approach was used to test for differences in the 1/*f* aperiodic parameters (slope and offset) in both the 3back and resting data. However, because these measures only provided a single value at each electrode (rather than a value for each frequency and timepoint), only the primary threshold RMS and TANOVA tests were used to assess these measures. Finally, the analyses of oscillatory activity after controlling for the aperiodic activity provided values for each electrode and frequency (but were averaged across timepoints). Thus, to analyse the aperiodic corrected oscillatory activity, we again used cluster-based statistics to test for clusters of significant frequencies (ignoring the temporal dimension since these measures were computed across the 500–900 ms range, or a 2 second period in the resting data).

## Results

### EEG oscillatory activity

The cluster-based statistics testing for differences in RMS at each frequency (3–45 Hz) and timepoint (0–1000 ms around the stimulus presentation time) indicated a significant main effect of time (*p* = 0.002), with a significant cluster that spread from 8 to 45 Hz and lasted from approximately 330 to 800 ms for frequencies within the alpha range, from 500 to 800 ms for frequencies in the beta range, and from 550 to 820 ms in the gamma range (see Figure 1). Post hoc analyses indicated that this result was driven by stronger power in the post-dosing EEG session for all of these frequency bands (reported below). There was no significant interaction in the RMS test between group and session (*p* = 0.299), nor main effect of group (*p* = 0.683). Similarly, there were no between session differences detected in the normalised TANOVA (*p* = 0.946), nor a significant interaction between drug and session (*p* = 0.724), nor significant main effect of drug (*p* = 0.665). These TANOVA results show that there were no differences in the topographical distribution of activity for the pre-dosing to post-dosing EEG session in baseline corrected frequency power after normalising for the global neural response strength, indicating that although there was an increase in global frequency power, the same source brain regions generated the activity.

**Figure 1.**
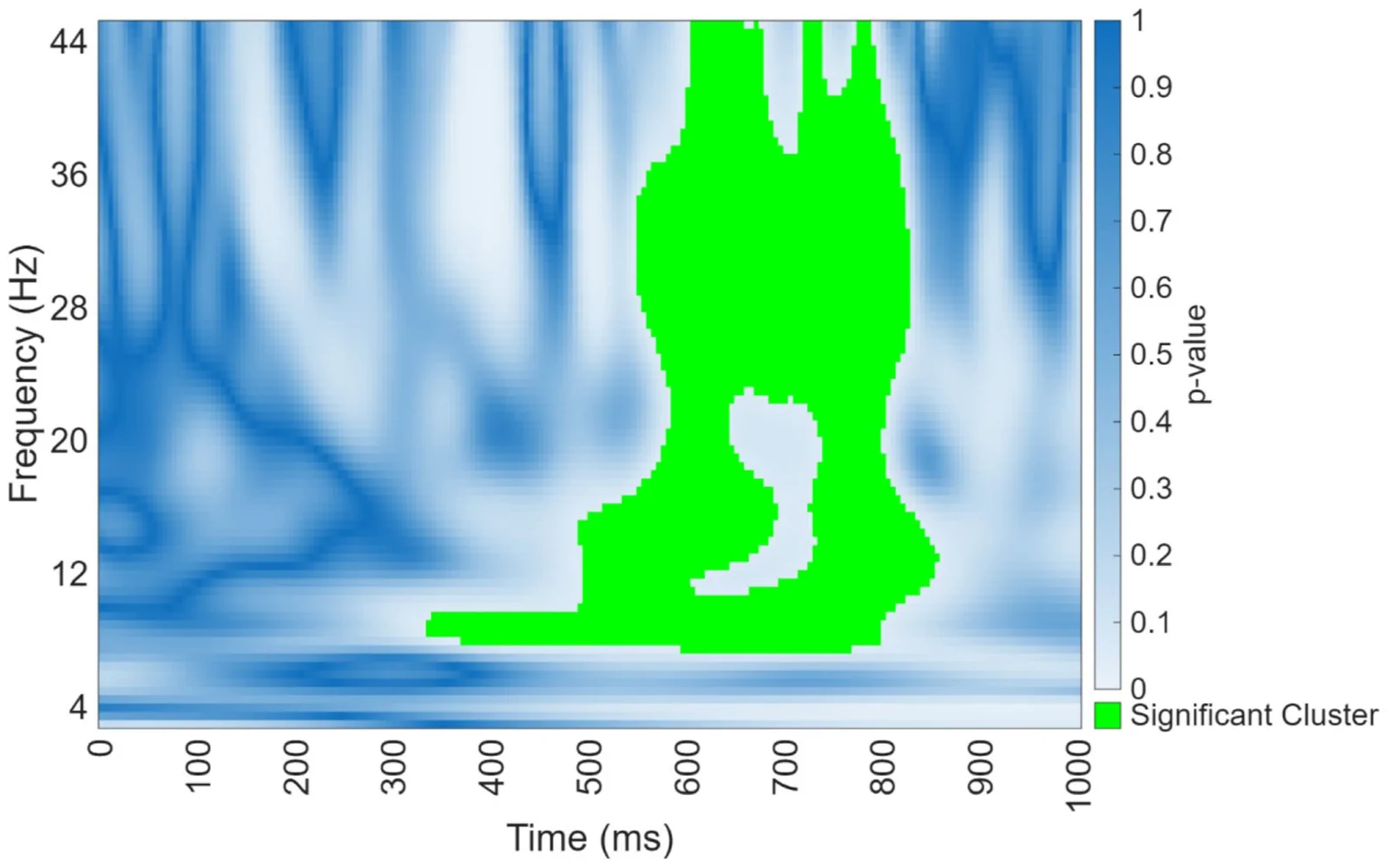
A *p*-value map of the comparisons between the baseline and post-dosing EEG session recordings, with permutation statistics conducted for each cell in the time-frequency matrix, followed by cluster-based statistics to determine if the size of the significant cluster exceeded the size of the significant clusters in the permutation shuffles of the data labels. A cluster of time-frequencies that passed both our initial *p*-value threshold and our cluster-based statistics threshold is highlighted in green. EEG: electroencephalography.

To explore the global differences in frequency power demonstrated by the RMS test, we undertook post hoc tests by extracting the mean RMS frequency power values averaged across the timepoints and frequencies within the significant cluster. We analysed these values using a repeated measures ANOVA in JASP. This analysis indicated a significant main effect of session with the post-dosing EEG session showing higher power than the pre-dosing EEG session with a large effect size: *F*(1,56) = 9.124, *p* = 0.004, ω^2^_*p*_ = 0.015, BF_10_ = 6.178. The analysis also indicated a significant interaction between session and drug with a medium-large effect size: *F*(1,56) = 7.267, *p* = 0.009, ω^2^_*p*_ = 0.012, BF_10_ = 5.053 (see Figure 2). Post hoc *t*-tests indicated that the interaction was driven by a difference between the pre-dosing EEG and post-dosing EEG session for the MDMA condition: *p*-holm < 0.001, Cohen’s *d* = 0.496, BF_10_ = 45.70, while the difference between sessions for the psilocybin condition was not significant: *p*-holm = 1.0, Cohen’s *d* = −0.028, BF_10_ = 0.203.

**Figure 2.**
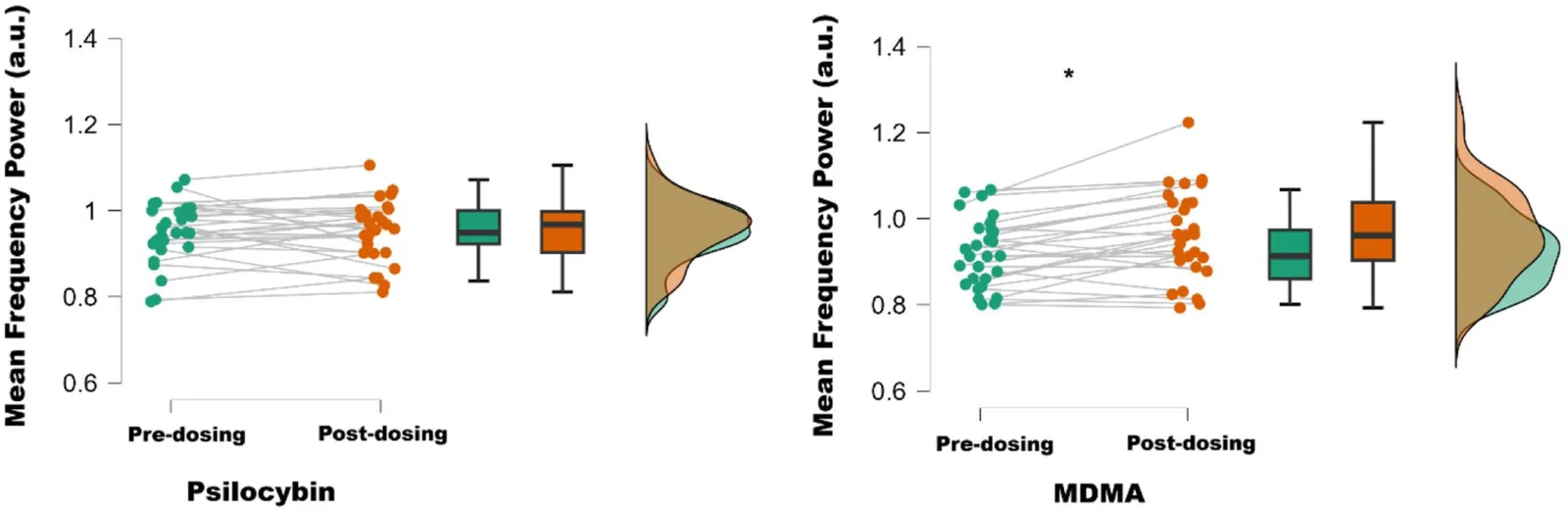
Frequency power averaged within the significant cluster for each of the conditions at each session. *p-holm < 0.01.

Exploratory analyses of the different frequency bands separately showed the same direction of effect in all of the frequencies across the cluster for the MDMA condition, but no significant effects for the psilocybin condition. For MDMA, all frequency bands showed significant differences in baseline corrected power between the pre-dosing and post-dosing EEG session, with the effects being strongest for the upper beta and gamma frequencies: alpha (8–12 Hz): *t*(28) = 2.293, *p* = 0.030, Cohen’s *d* = 0.426, BF_10_ = 1.848, lower beta (12–18 Hz): *t*(28) = 2.141, *p* = 0.041, Cohen’s *d* = 0.398, BF_10_ = 1.416, upper beta (18–29 Hz): *t*(28) = 3.153, *p* = 0.004, Cohen’s *d* = 0.585, BF_10_ = 10.338, gamma (29–45 Hz): *t*(28) = 3.909, *p* < 0.001, Cohen’s *d* = 0.726, BF_10_ = 58.001. For psilocybin, no significant differences in baseline corrected power were present for any of the separate frequency bands: alpha (8–12 Hz): *t*(28) = 0.238, *p* = 0.814, Cohen’s *d* = 0.044, BF_10_ = 0.203, lower beta (12–18 Hz): *t*(28) = 0.484, *p* = 0.632, Cohen’s *d* = 0.090, BF_10_ = 0.220, upper beta (18–29 Hz): *t*(28) = 0.283, *p* = 0.780, Cohen’s *d* = 0.052, BF_10_ = 0.205, gamma (29–45 Hz): *t*(28) = −0.096, *p* = 0.924, Cohen’s *d* = 0.018, BF_10_ = 0.198.

We note that baseline corrected frequency power showed values of <1 after stimulus presentation, indicating that frequency power briefly increased following stimulus presentation, then showed a prolonged decrease relative to before the presentation of the stimulus, with this decrease being smaller in the post-dosing EEG session (see Figure 3). This finding is interesting in the context of previous research, which has indicated that higher frequency power is related to WM and executive functions, and better performance in the 3back task. Combined with findings reported by previous research, the current result may suggest that frequency power increases immediately following stimulus presentation, enabling sensory processing and encoding of the stimuli, followed by a decrease in power that gradually returns to higher levels approximately 1 second after the stimulus (and 1 second prior to the next stimulus). The higher frequency power values 1–2 seconds after stimulus presentation may reflect the engagement of executive function processes to rehearse the current stimulus, update WM storage, and maintain the temporal ordering of stimuli in memory.

**Figure 3.**
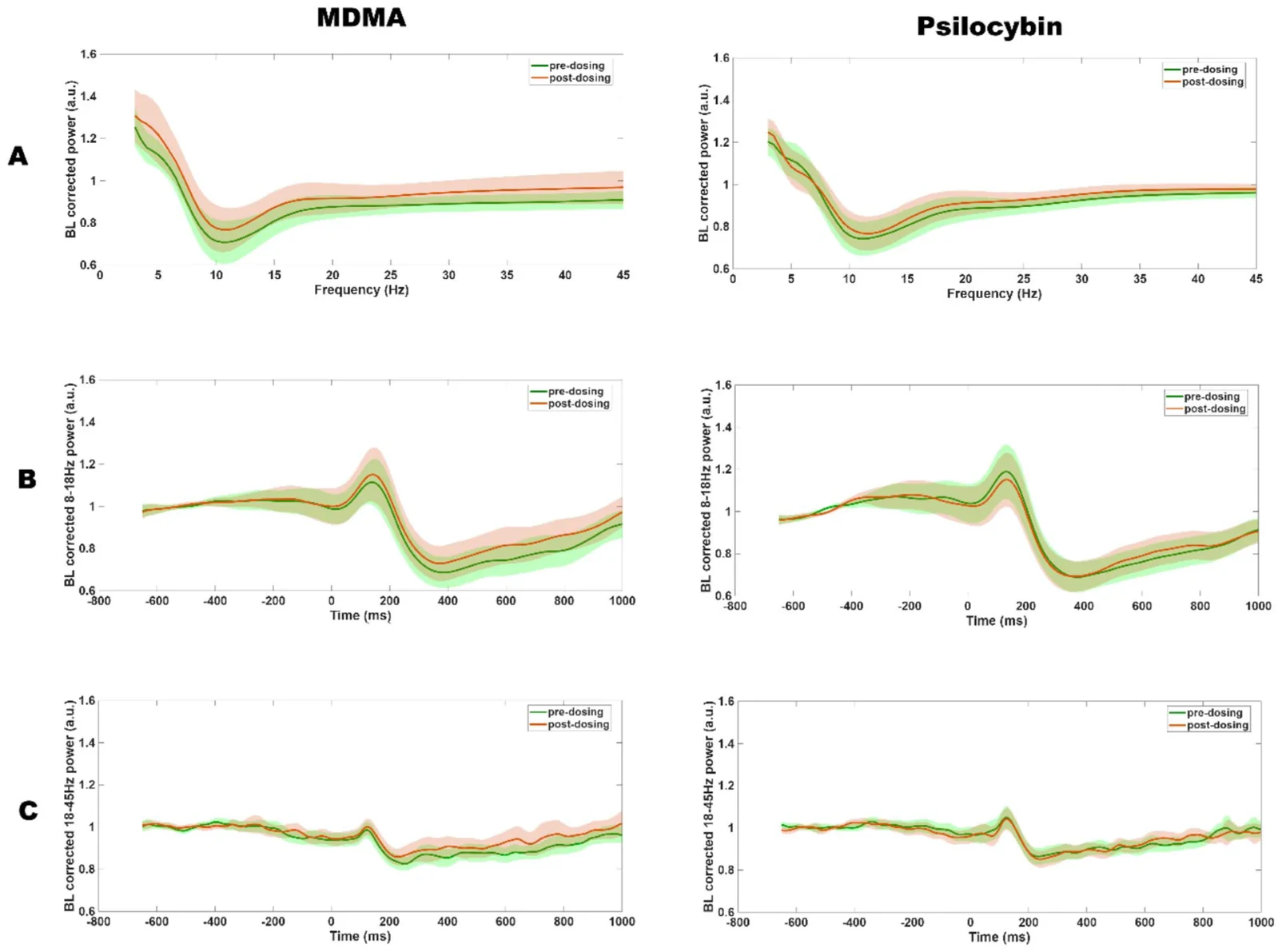
Baseline corrected frequency power. (a) Averaged across the 600–800 ms period where all frequencies above 8 Hz showed a significant difference from pre-dosing to post-dosing EEG session; (b) averaged across the alpha to lower beta frequencies (8–18 Hz) at each timepoint in the epoch, and (c) averaged across the upper beta to gamma frequencies (18–45 Hz) at each timepoint in the epoch. The shading reflects 95% confidence intervals. Note that frequency power decreases relative to the baseline period (−650 to −350 ms) in each epoch, but frequency power is also higher in the post-dosing EEG session compared to the pre-dosing session during the 600–800 ms period, particularly for the MDMA condition. MDMA: 3,4-methylenedioxymethamphetamine; EEG: electroencephalography.

### EEG aperiodic activity

Since the MDMA condition showed increases in frequency power from the pre-dosing to post-dosing EEG session with larger effect sizes for the higher frequencies, and acute psychedelic administration is suggested to affect the slope of the aperiodic activity, we considered the potential that our frequency power results were driven by changes in the slope of the aperiodic spectrum. Counter to this expectation, when the RMS of the aperiodic slope within the 500–900 ms period was compared between pre-dosing and post-dosing EEG sessions, the post-dosing EEG session in the psilocybin condition showed a significantly steeper slope: RMS permutation test: *p* = 0.007, *t*(28) = 2.501, *p* = 0.018, Cohen’s *d* = 0.464, BF_10_ = 2.721 (see Figure 4). This result was driven by a global effect (across all electrodes) as the psilocybin condition did not show a significant difference in the distribution of the 1/*f* slope independent of global differences in amplitude: normalised TANOVA permutation test *p* = 0.996. However, we note that when we inspected the maximum differences across electrodes, the differences were largest in the occipital electrodes, with the comparison averaged across O1, O2, and Oz showing: *t*(28) = 3.925, *p* < 0.001, Cohen’s *d* = 0.802, BF_10_ = 155.138. The MDMA condition did not show a significant difference between the sessions in the RMS or TANOVA tests: RMS permutation test: *p* = 0.176, *t*(28) = 0.976, *p* = 0.337, Cohen’s *d* = 0.181, BF_10_ = 0.305, nor a significant difference in the distribution of the 1/*f* slope independent of global differences in amplitude normalised TANOVA permutation test *p* = 0.324. No significant differences were detected in pre-dosing to post-dosing EEG session comparisons of the aperiodic offset for either psilocybin or MDMA (RMS: *p* = 0.616 and *p* = 0.439, respectively, TANOVA: *p* = 0.836 and *p* = 0.825, respectively).

**Figure 4.**
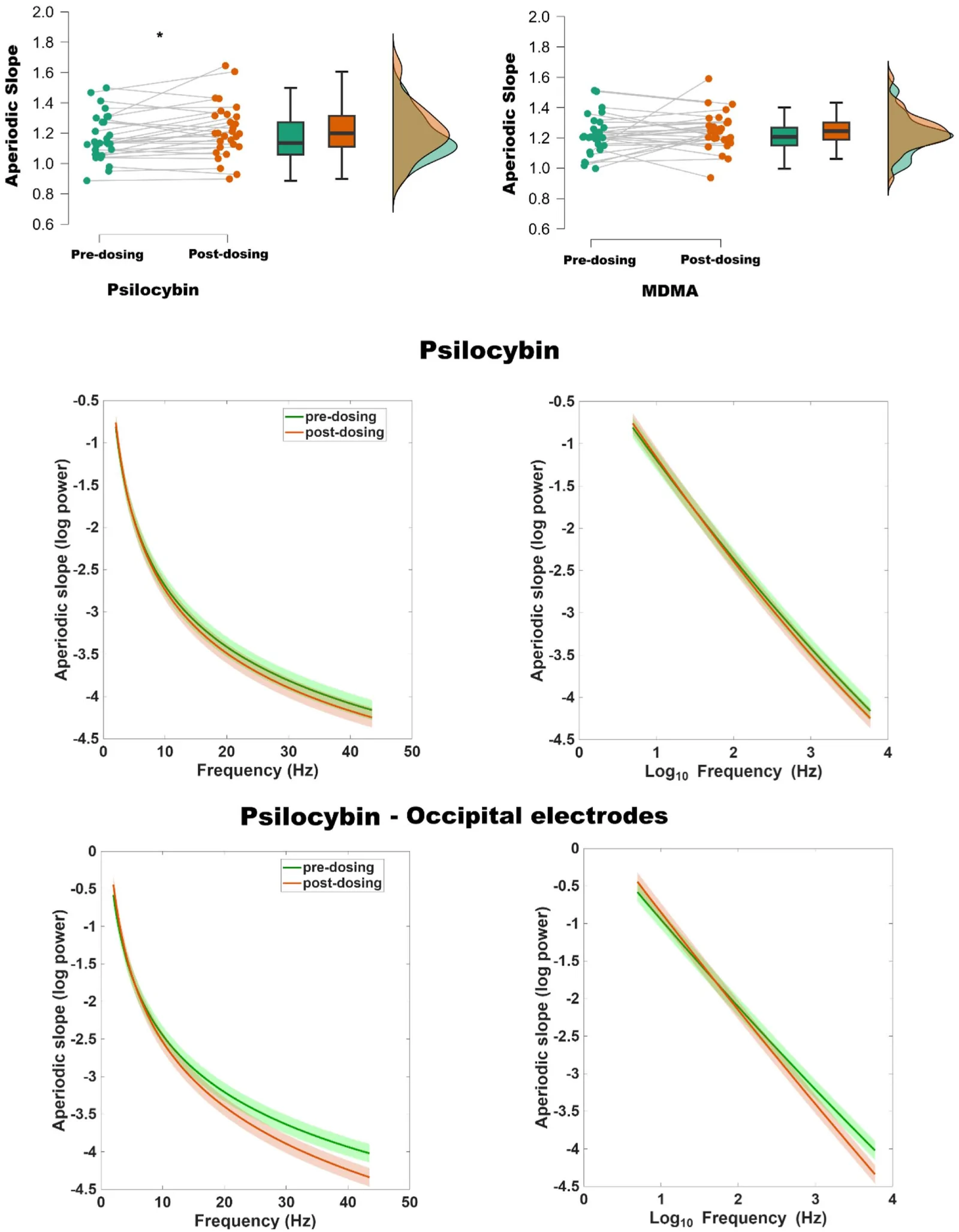
Top: raincloud plots depicting aperiodic slope values (where higher values represent steeper slopes) for each session and each drug separately (*p < 0.01). Middle: log_10_ power depicting the mean slope in the pre-dosing and post-dosing EEG session for the psilocybin group (with shading indicating 95% confidence intervals) for each frequency (left) or log_10_ frequency (right). Bottom: the same log_10_ power values but averaged across only the occipital electrodes. EEG: electroencephalography.

If we had not undertaken this test of the aperiodic slope, differences in the aperiodic slope could have been a reasonable explanation for an increase in power in the 8–45 Hz range. However, in the context of the increase in baseline corrected frequency power from 8 to 45 Hz within the same time window, these results suggest that the increased frequency power in the MDMA condition reflects increases in oscillatory power rather than being driven by a flattening of the aperiodic spectrum. Counter to this potential explanation, the aperiodic results suggest a steepening of the aperiodic spectrum in the psilocybin condition, but no change in the MDMA condition.

### Oscillatory power after correcting for aperiodic activity

The analysis reported in the previous section suggested that the aperiodic slope was steeper in the post-dosing EEG session for psilocybin. We considered that this may have masked differences in baseline corrected oscillatory activity, as a steeper aperiodic slope is associated with lower overall frequency power in the alpha to gamma frequencies (see Figure 4). Additionally, we note that potential differences in the baseline period’s frequency power (for example, differences that lasted the entire epoch) were undetectable by our analysis of baseline corrected frequency power, as differences in the baseline period would be transposed into the rest of the data by the baseline correction (see Bailey et al., 2023c for an example of this in event-related potential data). Thus, we undertook an analysis of oscillatory power after correcting for the aperiodic activity (by subtracting the *fooof* estimated aperiodic activity from the full power spectrum to obtain an estimate of just the oscillatory activity).

When correcting for the aperiodic slope (but not baseline power), the MDMA condition showed a cluster of significantly stronger delta power in the post-dosing EEG session (from 1 to 1.75 Hz, *p* < 0.001), theta power in the post-dosing EEG session (from 4.5 to 6.25 Hz, *p* < 0.001), and a cluster of significantly stronger alpha power in the post-dosing EEG session (10.75–14.25 Hz, *p* < 0.001; see Figure 5). Similarly, the psilocybin condition showed clusters of significantly stronger upper theta to alpha power in the post-dosing EEG session (6–10.5 Hz, *p* < 0.001), significantly stronger upper alpha to lower beta power in the post-dosing EEG session (12.5–16.75 Hz, *p* < 0.001), and significantly stronger beta power in the post-dosing EEG session (24.5–25.25 Hz, *p* < 0.001; see Figure 5). There were no significant differences in the TANOVA for either drug (all *p* > 0.05).

**Figure 5.**
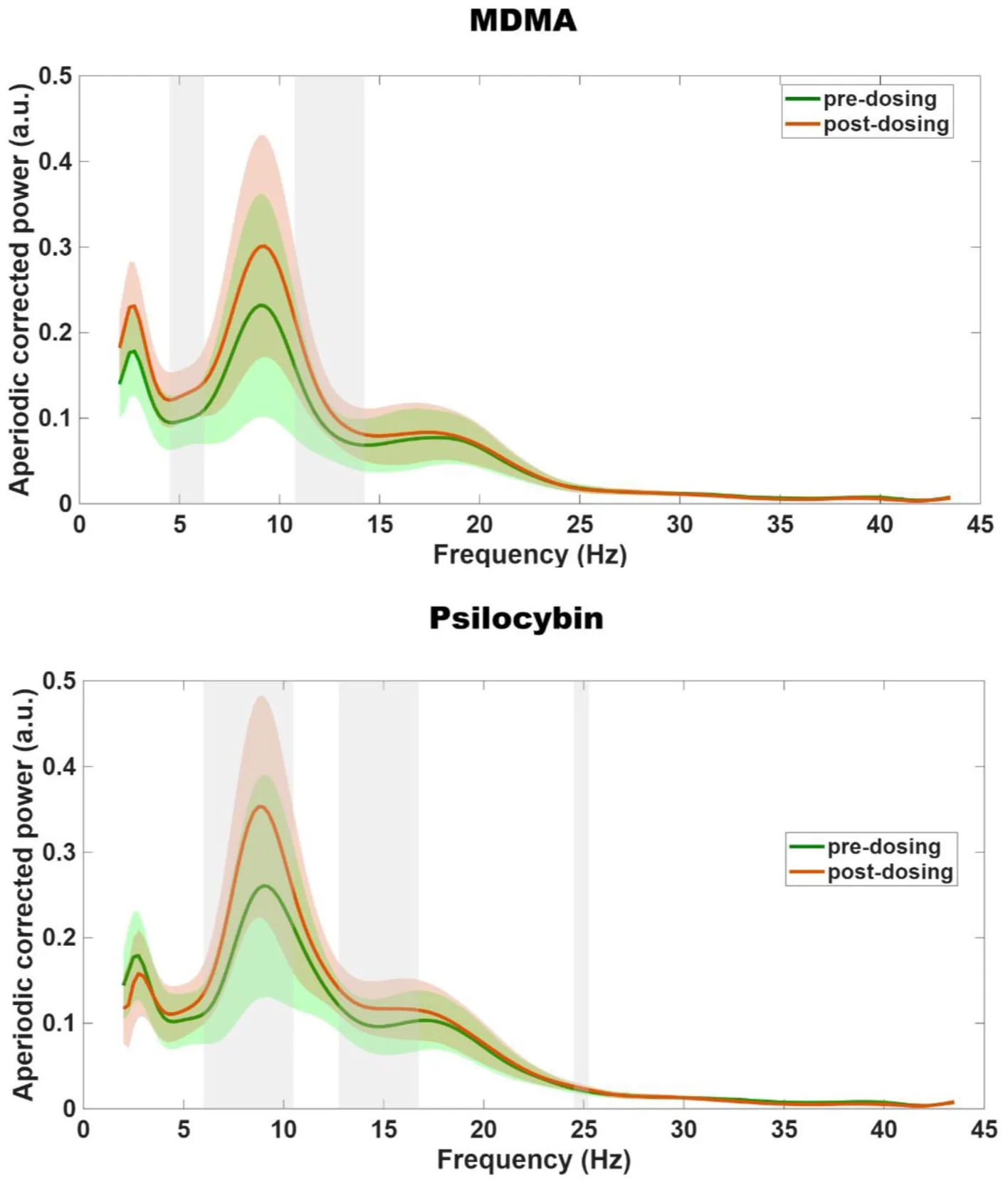
Frequency power in the 500–900 ms period following correct non-target stimuli, after correction for the aperiodic activity (shaded error bars indicate 95% confidence intervals, and grey shaded sections indicate frequency clusters that significantly differed between the conditions for a broader spread of frequencies than 95% of the permutations).

Finally, to assess whether either oscillatory activity or changes in oscillatory activity related to task performance (3back accuracy or changes in 3back accuracy), we performed Pearson’s correlations between the mean aperiodic corrected RMS theta activity (4–8 Hz) and RMS alpha activity (8–13 Hz). None of the neural activity measures or pre-dosing to post-dosing EEG session changes in these measures correlated with 3back accuracy or changes in 3back accuracy (all *p* > 0.05).

### Resting state aperiodic slope and oscillatory power

Our analyses of the resting state aperiodic slope indicated a trend towards a steeper aperiodic slope in the psilocybin condition at the post-dosing EEG session compared to pre-dose (*p* = 0.066). Given the cortical activations required during the 3back task, we suspected EEG data during the 3back task might have had a better chance of eliciting global changes in activity that would have a better chance of detection with our statistical tests (which included all electrodes). To assess the potential that the same effects were present in our resting data, but were more spatially focal, we examined the 3back task data to determine which electrodes showed the largest differences in aperiodic slope (O1, O2, and Oz). We then repeated statistical tests in our resting data, focused on the averaged aperiodic slope from those electrodes. This analysis showed that within the psilocybin condition, the eyes-closed resting post-dosing EEG session showed a significantly steeper aperiodic slope at these occipital electrodes: *t*(28) = 2.848, *p* = 0.008, Cohen’s *d* = 0.529, BF_10_ = 5.418 (see Figure 6). Analyses of the eyes-closed resting oscillatory activity after controlling for the estimated aperiodic activity showed no significant pre- to post-dosing EEG session differences for either condition (all *p* > 0.05).

**Figure 6.**
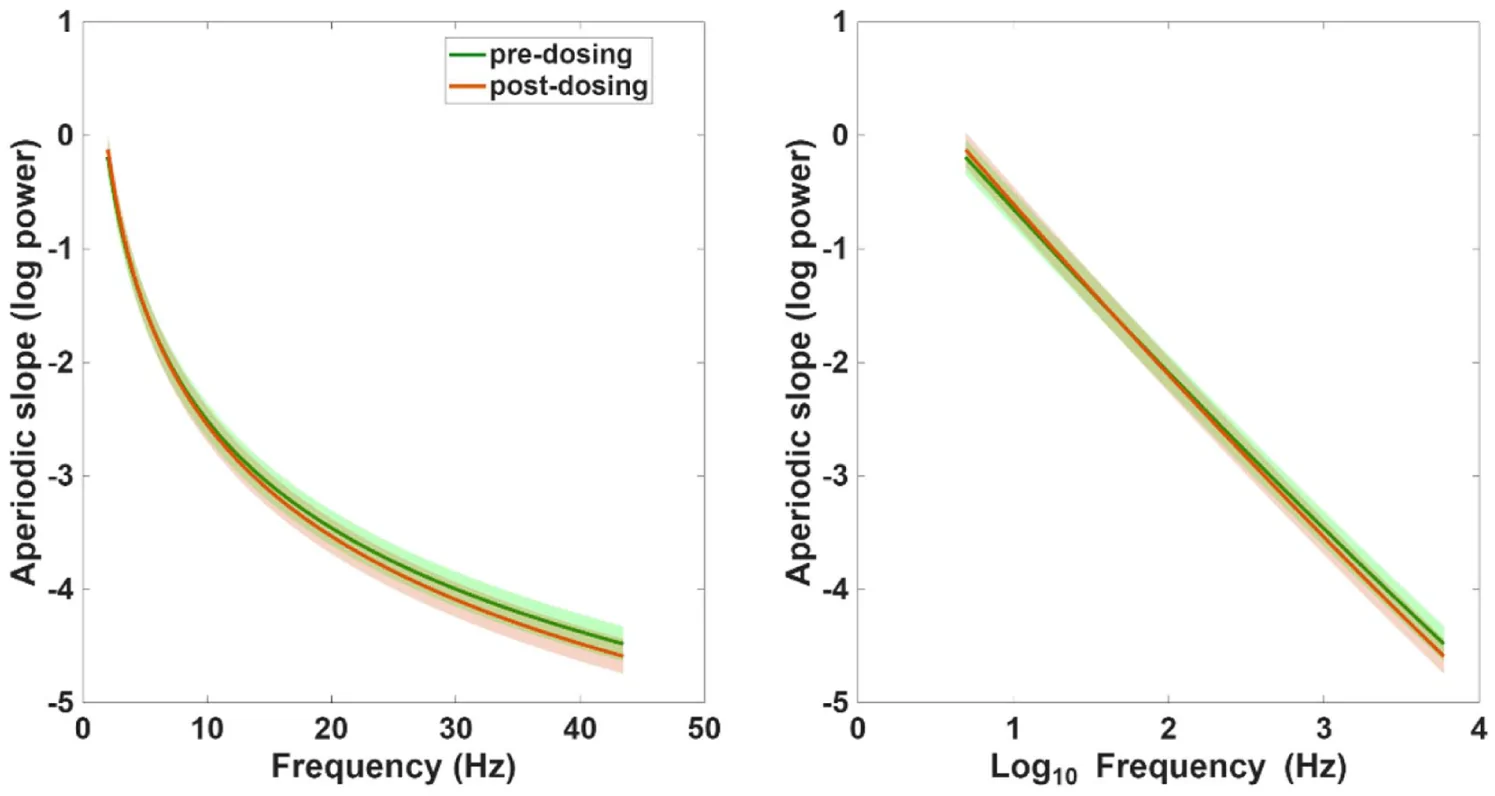
Root mean squared (across all electrodes) log_10_ power depicting the mean slope from occipital electrodes in the pre-dosing and post-dosing EEG session for eyes-closed resting data within the psilocybin group (with shading indicating 95% confidence intervals) for each frequency (left) or log_10_ frequency (right). Globally (using the RMS across electrodes), results showed a trend towards a significantly steeper slope in the post-dosing EEG session (*p* = 0.066), with this reflecting the same pattern as the 3back data. When the same analysis was conducted restricted to occipital electrodes (which showed the strongest effect for aperiodic slope in the 3back data), the result was significant (*p* = 0.008). EEG: electroencephalography; RMS: root mean squared.

### Behavioural data

For our primary (a priori planned) analysis of the 3back behavioural data, comparisons of accuracy between pre-dosing and post-dosing EEG session showed a significant effect of session for MDMA: *t*(28) = 2.567, *p* = 0.005, Cohen’s *d* = 0.561, BF_10_ = 7.809. However, no significant effect of session was present for psilocybin: *t*(28) = 1.543, *p* = 0.134, Cohen’s *d* = 0.287, BF_10_ = 0.570 (see Figure 7).

**Figure 7.**
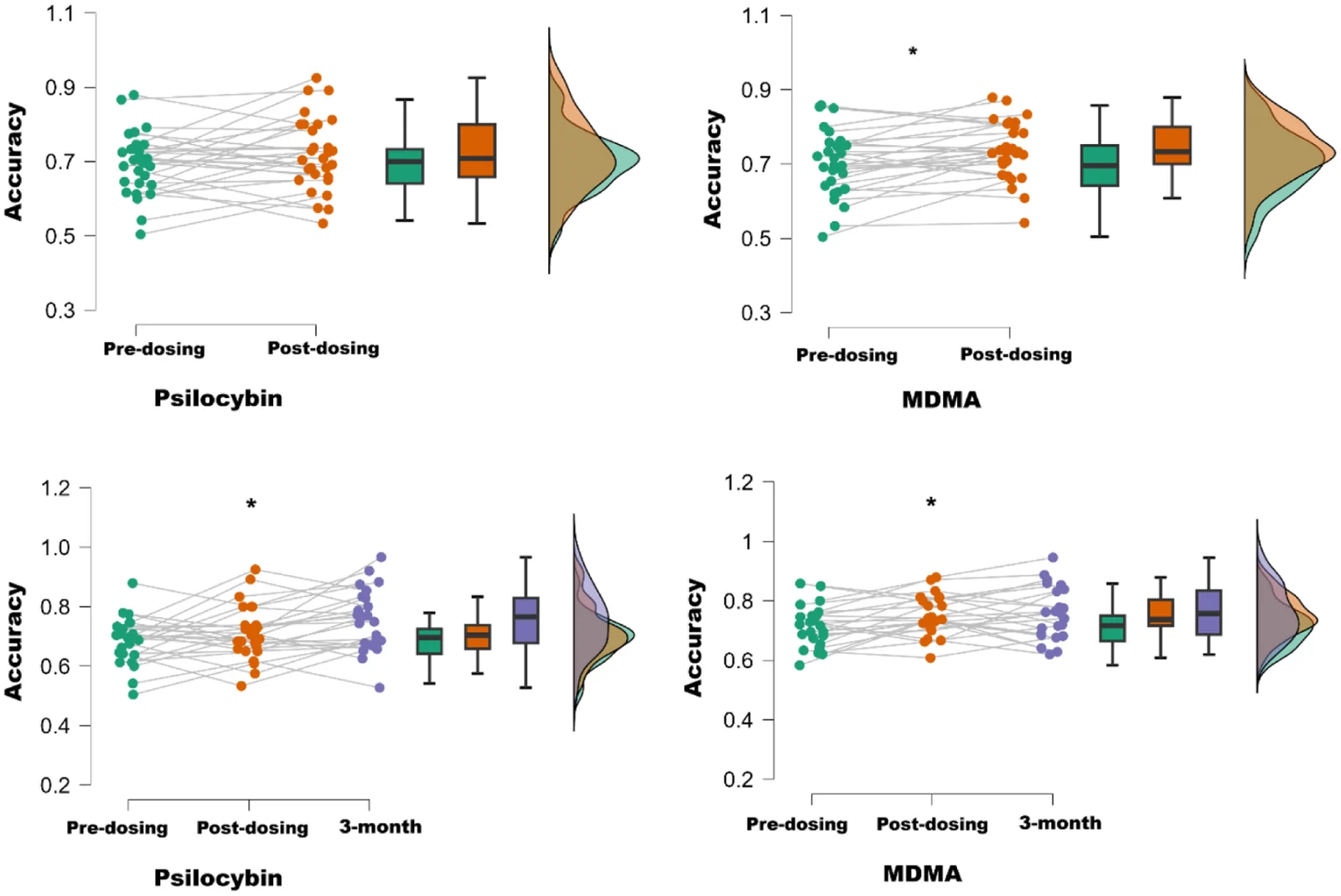
3back task accuracy in the different measurement sessions for each drug. Top: data from only the pre-dosing and post-dosing EEG sessions, including all participants who provided data for both sessions. Bottom: data from the pre-dosing, post-dosing EEG session and 3-month follow-up sessions, including only the participants who attended all sessions. EEG: electroencephalography. *p < 0.01.

Since our study did not include a control arm, we performed additional analyses to explore whether the significant improvement in WM accuracy might be driven by practice effects from repeating the task. We reasoned that similarity in performance trajectories across the two drug groups would be consistent with a practice effect account, while differential trajectories would be difficult to attribute to practice effects alone. However, we also note that neither pattern is fully diagnostic in the absence of a control group, as differences between groups could also reflect genuinely distinct pharmacological effects of MDMA and psilocybin on task performance. Thus, these analyses should be interpreted as exploratory, with further research including a control group required to verify any tentative conclusions we might draw here. First, we compared the two groups using a repeated measures ANOVA, effectively using the psilocybin group as a control group for the MDMA group. This analysis showed a main effect of session with a very large effect size: *F*(1,56) = 9.066, *p* = 0.004, ω^2^_*p*_ = 0.027, BF_10_ = 9.280, but no main effect of group: *F*(1,56) = 0.353, *p* = 0.555, ω^2^_*p*_ = 0.000, BF_10_ = 0.397 or interaction between group and session: *F*(1,56) = 0.247, *p* = 0.621, ω^2^_*p*_ = 0.000, BF_10_ = 0.288. When the 3-month follow-up session was included in the repeated measures ANOVA (which entailed a reduced sample size due to 10 participants not completing the 3-month follow-up assessment), the analysis again showed a significant main effect of session: *F*(2,92) = 11.051, *p* < 0.001, ω^2^_*p*_ = 0.067, BF_incl_ = 503.596, but no main effect of group: *F*(2,46) = 1.338, *p* = 0.253, ω^2^_*p*_ = 0.007, BF_incl_ = 0.535, or interaction between session and group: *F*(2,92) = 1.138, *p* = 0.325, ω^2^_*p*_ < 0.001, BF_incl_ = 0.288 (see Figure 1). Post hoc *t*-tests indicated that the effect of session was due to significantly better accuracy in the post-dosing EEG session compared to pre-dosing session (*p*-holm = 0.014, Cohen’s *d* = 0.388, BF_10_ = 5.315), significantly better accuracy in the 3-month follow-up session compared to the pre-dosing session (*p*-holm < 0.001, Cohen’s *d* = 0.678, BF_10_ = 272.074), and significantly better accuracy in the 3-month follow-up session compared to the post-dosing EEG session (*p*-holm = 0.039, Cohen’s *d* = 0.289, BF_10_ = 1.299).

To further assess whether the likely practice effect was comparable in size to typical practice effects without an intervention, we computed the effect size from previous research that required 40 young Chinese adult participants (aged 18–25 years) to repeat a 3back task after a 3-week delay (Yeung and Han, 2023). This was achieved by computing effect sizes from their study using the means and standard deviation they reported, combined with the correlation between repeated measures from our research (since Yeung and Han did not report the repeated measure correlation). This provided Cohen’s *d* = 0.599 (95% confidence interval = 0.203–1.099, 90% confidence interval = 0.275–1.027). We note that this is very similar to the pre-dosing to post-dosing EEG session effect size shown by our MDMA group, and that the effect size for our psilocybin group also falls within the 90% confidence interval. While we acknowledge there are other differences between the two studies, we note that the younger age of the sample from Yeung and Han (2023) would likely be expected to be associated with stronger practice effects than the practice effects expected for our older sample. It is also worth noting that some research in healthy individuals has indicated a lack of practice effects for the 3back task when measured before and after a sham brain stimulation condition, even within a brief (15 minute) delay between tasks (Chung et al., 2018). As such, our results suggest that, at minimum, MDMA and psilocybin exposures did not disrupt typical practice effects for the WM task.

## Discussion

The present uncontrolled study explored the differences associated with single dose psilocybin and MDMA exposure with regards to neurophysiological activity during a WM task in healthy participants 5–16 days after administration. Our findings may suggest enduring effects of these substances when administered in controlled settings, revealing intriguing potential neuroplasticity-related changes that last beyond the acute phase of drug action. Our EEG analyses indicated enhanced oscillatory power during the 3back task in the post-dosing EEG session across a broad frequency range (from theta activity up to gamma activity) for both drugs. Our analysis also indicated a steeper aperiodic slope globally following psilocybin during the 3back task, with the effect strongest in the occipital electrodes, a pattern that was also present in the eyes-closed resting post-dosing EEG session. Concurrent with these neurophysiological effects participants showed improved accuracy in a 3back task compared to their performance prior to the dosing session, with better accuracy post-dosing EEG session for MDMA, and at the 3-month timepoint for both drugs. However, we note that the uncontrolled nature of our study limits our ability to conclude that these effects were related to the drug exposure. Thus, we present our study protocol as a template for how to undertake rigorous research into the effects of psychedelics on neural activity, with the recommendation that future research include an appropriate control condition to enable stronger conclusions.

As far as we are aware, this is the first study to compare WM-related EEG activity before and after a single dose of psilocybin or MDMA, and these potentially enduring effects of psychedelics on neurophysiological activity have not previously been demonstrated. Both substances were associated with significant post-dosing EEG session increases in WM-related oscillatory power across multiple frequency bands, from theta through to gamma frequency ranges. Power increases from the alpha to gamma frequency range were present for baseline corrected frequency power in the MDMA condition, and for both drugs in the theta to upper alpha range when oscillatory power was isolated by subtracting the frequency power from a modelled estimate of the aperiodic activity. The psilocybin group additionally showed a steeper aperiodic activity 1/*f* slope after the dosing session in both the 3back task (across all electrodes, with the strongest effect in the occipital electrodes), and during eyes-closed resting (with significant differences present in occipital electrodes). We note that it is possible that the changes in oscillatory neural activity were due to practice effects, with repeated exposure to the task enabling participants to modify their neural activity to produce better task performance. However, the consistency of the changes in the aperiodic slope in the psilocybin condition across both eyes-closed resting and the 3back task suggest an enduring effect that is not related to task practice effects (since it is unlikely that practice effects would also translate to the eyes-closed resting EEG recordings).

Interestingly, the finding that exposure to psilocybin was associated with a steepening of the aperiodic slope contrasts with previous research on acute neurophysiological changes associated with psychedelics. That previous literature indicates that acute serotonergic psychedelic exposure typically flattens the aperiodic slope (although we are not aware of this finding having been demonstrated specifically for psilocybin; Godfrey et al., 2025). This aperiodic flattening during acute exposure may be indicative of a shift towards neural excitation and away from inhibition, or of the disruption to regular neural depolarisation organisation (Godfrey et al., 2025). This conflict between the acute effects and non-acute effects requires exploration. One possible explanation is that the changes were driven by post-dosing EEG session neuroplastic compensation effects, whereby homeostatic neural mechanisms were engaged to counter the acute effects, with engagement of these mechanisms lasting until the post-dosing EEG session 5–16 days later. Alternatively, psilocybin administration has been suggested to provoke neuroplasticity, showing a strong affinity for binding to a brain-derived neurotropic factor receptor (Moliner et al., 2023). Emerging theories also suggest that psychedelics promote neuroplasticity through mechanisms including increased dendritic branching, synaptogenesis, and enhanced neural connectivity (Vollenweider and Preller, 2020). It may be that the steeper aperiodic slope in the post-dosing EEG session is driven by these neuroplastic changes, with perhaps the neuroplastic effects predominantly influencing inhibitory connections in the post-acute period. We note that serotonergic psychedelics bind to 5-HT2A receptors, producing an excitatory effect on layer 5 pyramidal neurons (Burt et al., 2021; Stoliker et al., 2025). This receptor is most densely expressed in the visual and frontal regions of the human brain (Burt et al., 2021), aligning with our finding that the effects of psilocybin on the aperiodic slope were strongest in the occipital electrodes.

Research has also shown that steeper aperiodic slopes in frontal brain regions are likely to be important for, and associated with, performance in WM and executive function tasks (Virtue-Griffiths et al., 2025), which may suggest the steeper aperiodic slopes we observed in the post-dosing EEG session (which included frontal regions) could provide functional benefits during WM. However, we note that neither the aperiodic slopes nor changes in the slopes correlated with 3back accuracy in our study, and the slope differences were strongest in occipital regions rather than frontal regions. Previous research has also indicated that steeper aperiodic slopes, reflecting functional inhibition, are observed in visual process brain regions after visual stimulation has ceased (Manyukhina et al., 2024). We note that the steeper aperiodic slopes we observed in the 3back task were detected immediately following the cessation of stimulus presentation in each trial (we measured the aperiodic slope during a 500–900 ms period, with the stimuli presentation occurring from 0 to 500 ms during each trial). Thus, it is possible that the steeper aperiodic slopes we observed in our 3back and eyes-closed resting post-dosing EEG sessions reflects increased inhibition within the visual brain regions during periods where no visual stimuli were presented. Further research is required to determine the functional implications of this steepening of the aperiodic slope.

Our finding of increased WM-related alpha activity in the post-dosing EEG session also contrasts with non-task-related effects reported during acute administration recordings, where a robust elimination of alpha oscillations is typically observed across psychedelic substances, including psilocybin (Godfrey et al., 2025). This disruption during acute psilocybin administration is suggested to relate to the disruption of default mode activity (Godfrey et al., 2025; Tyls et al., 2016), and to be driven by activation of 5-HT2A receptors (Kometer et al., 2013). These acute effects have been shown to last up to 24 hours (Nikolic et al., 2024). Theta and beta decreases are also consistently reported during acute administration, effects which are similar across a range of psychedelic substances (Godfrey et al., 2025). However, our results at least partially align with those reported in previous research on the non-acute effects of recreational MDMA use on EEG activity, which indicated that the frequency of recreational MDMA use correlated positively with eyes-closed resting state frequency power in the alpha (8–12 Hz) and beta (12–20 Hz) bands (although in contrast to our results, not with the theta (4–7 Hz) band) (Dafters et al., 1999). Cross-sectional research has also indicated higher levels of resting theta and alpha power in heavy polydrug ecstasy users compared to low and non-users (Adamaszek et al., 2010). Enhancements to auditory evoked theta power 2 weeks following psilocybin-exposure (compared to both pre-dosing measurement, and a placebo condition) have also been reported, a result which correlated with improvements in depression symptoms, was suggested by the authors to reflect potential neuroplastic change related to the psilocybin (Skosnik et al., 2023). However, it is worth noting that the differences in oscillatory activity we observed were restricted to the 3back task, whereas previous research has predominantly examined resting recordings. Our results might suggest that examining neural activity linked to specific cognitive processes might provide increased power to detect potentially enduring effects of psychedelics on neural activity.

Previous research has shown that oscillatory power in these bands is associated with both performance in WM and executive function tasks (Gevins and Smith, 2000; Tallon-Baudry et al., 1998). However, we note here also that our measures of alpha and theta power, and changes in these measures (after controlling for aperiodic activity), did not correlate with 3back accuracy in our study. Additionally, since no differences were detected in these measures during the eyes-closed resting recordings, we cannot rule out the potential that practice effects from the repeated presentation of the 3back tasks drove differences in neural activity. Nonetheless, our neural activity measures suggest a lack of adverse effects on neural activity, with changes to the underlying neurophysiological processes that support WM, executive function, and learning maintained over extended time periods from a single dose exposure to psilocybin or MDMA, in alignment with the improved behavioural performance we detected.

In addition to our neurophysiological findings, it was interesting to observe that WM accuracy was improved in the post-dosing EEG session for both drugs. This result stands in marked contrast to previous literature suggesting likely cognitive deficits associated with recreational MDMA use (Montgomery and Roberts, 2022; Roberts et al., 2016). The improved accuracy shown in our results is perhaps likely to reflect a practice effect, with the effect size of the accuracy improvement showing a similar magnitude to practice-related improvements reported by previous research (Yeung and Han, 2023). This suggests that although our study design cannot indicate improved WM performance from MDMA or psilocybin, at a minimum, our results provide evidence against a WM impairment from the single exposure to MDMA. We note that studies reporting MDMA-related cognitive impairments have typically examined individuals with histories of chronic, uncontrolled recreational use, often involving higher doses, frequent administration, and with additional confounds of polydrug use and the potential adulterants often found in non-pharmaceutical preparations of MDMA, as well as reductions in sleep associated with recreational use (Gouzoulis-Mayfrank and Daumann, 2006; Montgomery and Roberts, 2022). Thus, our result may suggest that cognitive impairments previously attributed to MDMA may result from factors associated with recreational use patterns rather than the pharmacological properties of pure MDMA itself. Similarly, our results provide evidence against cognitive impairment from a single exposure of psilocybin, and our findings of improved accuracy at the 3-month follow-up timepoint perhaps also provides support for suggestions that psilocybin may promote cognitive function (Meshkat et al., 2024).

Overall, our results align with the growing body of evidence suggesting that psychedelics are not associated with persistent impairments in cognitive function when used in controlled settings. This finding is particularly important given its growing application in psychedelic assisted therapy. The neurophysiological changes observed in both groups may be of interest within the wider corpus of research on psychedelic mechanisms; however, given the absence of a reliable relationship between the neurophysiological and behavioural findings in the present study, we are unable to draw direct conclusions regarding their functional or therapeutic significance. Establishing whether such neurophysiological changes are meaningfully related to cognitive or therapeutic outcomes will require future studies with sufficient power to examine brain-behaviour relationships directly. The persistence of neurophysiological differences at the post-dosing assessment is notable and consistent with accounts of drug-induced neuroplastic adaptation; however, we acknowledge that in the absence of a control group, a contribution from repeated task engagement cannot be ruled out. This observation should therefore be interpreted cautiously and treated as a hypothesis for future controlled investigation rather than as direct evidence of lasting neuroplastic change.

Some caveats to our conclusions are warranted. Given the lack of a control group, we are unable to determine whether the oscillatory and behavioural effects may simply reflect practice effects or task familiarity effects, so cannot conclude that the drugs were responsible for the altered WM performance and neural activity. We note challenges regarding the inclusion of an appropriate control group are faced across psychedelic research and are not unique to this study (Nayak and Zahid, 2026). Additionally, the inclusion of multiple active conditions and multiple measurement timepoints in the present design provides at least some leverage against this interpretation (Nayak and Zahid, 2026). Practice effect research indicates that the trajectory of practice effects from repeated task exposure shows the largest gains between first and second task exposure, which subsequently plateaus (Bartels et al., 2010); the persistence of effects at the 3-month follow-up may therefore be more consistent with a sustained drug-related change than with familiarity alone. Furthermore, MDMA and psilocybin have distinct pharmacological profiles and mechanisms of action, which may have led to the differential patterns of change in both neural activity and behavioural performance that we observed between the drugs. In contrast, a pure practice or task familiarity effect would be expected to produce broadly similar improvements across both groups regardless of the drug. Thus, it is possible that MDMA’s well-documented effects on serotonin, dopamine, and norepinephrine systems may have facilitated learning or consolidation processes that enhanced subsequent task performance. Our 3-month follow-up results also align with suggestions that psilocybin may hold the potential to improve cognitive function (Meshkat et al., 2024).

Furthermore, the changes we observed in the aperiodic slope following psilocybin were consistent across the WM task and eyes-closed resting, suggesting an enduring neurophysiological change not explainable simply by practice effects. There is a growing body of evidence suggesting that psilocybin in particular may initiate neuroplastic processes that persist over weeks to months following a single dose, holding the potential for cumulative gains in function during that time period (e.g., Shao et al., 2021). Under this framework, continued improvement at 3 months is consistent with a drug-related interpretation. The trajectory of improvement observed here—modest at post-dosing, larger at 3 months—may therefore reflect the unfolding of sustained neuroplastic change rather than cumulative task exposure.

Notably, a comparison of the magnitude of observed behavioural improvements against published data for typical practice effects on this task suggests that, at minimum, neither compound impaired WM performance relative to expected retest gains (however, we note that the practice effect sizes reported from previous research were obtained from a population that did not match our population, with a different age range and cultural context). A mediation analysis examining whether neurophysiological changes mediated the observed behavioural improvements would have provided stronger causal evidence; however, the present sample was insufficiently powered to support such an analysis. This represents an additional limitation, and future work with larger samples should examine whether drug-induced neurophysiological changes mediate improvements in WM performance, which would provide more direct evidence of a neurobiological mechanism underlying the behavioural effects. Nonetheless, in the current study, the absence of a placebo or active control condition means that practice effects cannot be fully ruled out, and future studies should incorporate such controls to more definitively isolate drug-specific contributions to WM performance.

Further research including a plausible active control condition is recommended to explore whether the improved accuracy and enhanced neural activity in the post-dosing EEG session may indicate cognitive enhancements beyond practice effects from a controlled single dose exposure to these drugs. However, at minimum, we can conclude that it seems unlikely that a single session exposure to MDMA or psilocybin was associated with WM impairment. We also report this result from a sample that is not confounded by polydrug use, uncontrolled substance content and dosage, multiple exposures, reductions in sleep associated with recreational use, and potential negative expectation effects from participants and researchers due to fears about the harms of these drugs, confounds that are common in studies of the effects of MDMA on cognition (Gouzoulis-Mayfrank and Daumann, 2006; Montgomery and Roberts, 2022).

Additionally, we note that there was substantial variability in the timing of our post-dosing testing sessions (5–16 days following the dosing session). This variability was unavoidable due to the flexibility required to recruit and test the 48 participants who were typically employed full-time. However, this should be recognised as a limitation for the conclusions of our study, with the variability in timing perhaps affecting the extent to which neural activity and behavioural performance was influenced by the drug exposure in the post-dosing testing session, particularly given that the neurobiological and psychological effects of both MDMA and psilocybin are known to evolve over the days to weeks following administration. This may have obscured or diluted effects that may have been more clearly observed at a standardised timepoint. We additionally note that there was some variability in the doses administered within both drug conditions, with variability in whether participants in the MDMA arm received the supplementary dose, and within the psilocybin condition, doses were adjusted according to participant body weight. This approach is consistent with standard practice in clinical psychedelic research, where dose adjustment is intended to account for individual differences in drug metabolism and to produce a more equivalent pharmacological effect across participants, rather than to introduce uncontrolled variability. Nonetheless, the possibility that differences in total administered dose contributed to variance in outcomes cannot be fully excluded. A formal analysis of dose-related differences in outcomes was not feasible given our sample sizes, rendering any such comparison underpowered and likely non-informative. Future studies with larger samples should examine dose-response relationships systematically to better characterise the influence of dosing protocols on cognitive and neurophysiological outcomes.

A further limitation concerns the absence of a formal measure of expectation effects. Participants were aware of which compound they had received, and we did not collect data on participant expectations regarding the cognitive or therapeutic effects of the administered compound. Given the growing evidence that set and setting (including prior beliefs and expectations about psychedelic compounds) can influence both subjective and cognitive outcomes following administration, expectation effects cannot be ruled out as a contributor to the observed findings (Gukasyan and Nayak, 2022). The direction of any expectation bias is difficult to predict a priori, as positive expectations about cognitive enhancement could inflate apparent improvements, while uncertainty or concern about drug effects could attenuate them. Future studies should incorporate validated measures of participant expectancy to allow these effects to be examined and statistically controlled for, providing a cleaner estimate of the pharmacological contribution to any observed cognitive changes.

An additional complexity in the interpretation of these findings is that 16 participants received both drugs across separate dosing sessions (after a minimum of 3-month washout period, which matched our exclusion criteria that aimed to ensure participant’s baseline data would not be confounded by recent drug use). This limitation meant that for these participants, the second drug was administered in the context of prior drug exposure within the study. This introduces the possibility of order effects or carry-over influences that cannot be fully disentangled in the present dataset. While this approach enabled the sample sizes within each drug condition to be sufficient for the primary analyses (given the resource intensive screening of participants required for this study), readers should exercise caution in interpreting the results as fully independent between drug comparisons. Future studies should employ fully independent between-subjects designs, or counterbalanced crossover designs with sufficient washout periods and adequate power to test for order effects explicitly.

We note that our conclusions also pertain only to the doses administered in the study (80 mg plus an optional supplementary dose of 40 mg after 1 hour at the discretion of participants, the therapist, and medical doctor for MDMA, and 25 mg of psilocybin for participants under 90 kg and 30 mg for participants over 90 kg in the psilocybin condition). They also pertain only to the single dose of each drug, only to the pharmaceutical grade MDMA/psilocybin used in the study, and only to the therapeutic capsule in which the substances were provided to participants. Finally, the study population consisted of healthy volunteers with training in psychedelic assisted psychotherapy, so our findings may not generalise to clinical populations with psychiatric conditions. Given that both psilocybin and MDMA are primarily being developed for therapeutic applications in mental health, studies in relevant patient populations will be crucial for understanding their full clinical utility and safety profile. We also suggest that future research examine whether the effects we have reported generalise to other cognitive domains, and whether they predict therapeutic outcomes.

## Conclusions

This study provides reassuring evidence that single exposures to psilocybin and MDMA in controlled clinical settings do not impair WM function and may even enhance WM performance. The observed neurophysiological changes may suggest that both psilocybin and MDMA induce lasting modifications in neural activity that persist beyond the acute phase of drug action, with a steepening of the aperiodic slope in occipital electrodes after psilocybin likely to reflect a neurophysiological change unrelated to practice effects during the WM task. However, we note that this conclusion must be caveated with the awareness that our study did not include a control arm, so effects might not be attributable to the drugs. The results provide initial evidence that cognitive safety concerns should not impede therapeutic development. The observed neurophysiological changes may also provide potential insights into mechanisms underlying therapeutic efficacy, suggesting potential evidence for neuroplasticity-related executive function or WM enhancing effects that may contribute to their therapeutic potential, with future research including a control arm required to verify these associations. Perhaps the most important contribution of the present study lies in the rigour and quality of the sample and administration context. These aspects of our study have enabled us to conclude, at minimum, that it seems unlikely that a single session exposure to MDMA or psilocybin was associated with WM impairment. Critically, we report this finding from a sample that is free from the confounds that have complicated much of the existing literature on MDMA and cognition—including polydrug use, uncontrolled substance content and dosage, multiple exposures, sleep disruption associated with recreational use, and negative expectation effects arising from concerns about drug-related harm (Gouzoulis-Mayfrank and Daumann, 2006; Montgomery and Roberts, 2022). Dosing was conducted by expert practitioners under controlled conditions, with verified substance sourcing and careful participant screening. Aside from our lack of control group, this methodological standard provides an exemplar for the standard to which studies of this kind can be conducted, and we hope the present work serves as a useful template for future research in this field seeking to isolate the true cognitive effects of these compounds from the noise introduced by uncontrolled recreational contexts.

## Data Availability

The de-identified data that support the findings of this study are available from the corresponding author, NWB, upon reasonable request.
